# Post-TB lung function, quality of life, and radiographic findings in children

**DOI:** 10.5588/ijtldopen.24.0675

**Published:** 2025-07-09

**Authors:** G.L. Becker, H. Kisembo, Y. Sato, L.H. Wendt, H. Aanyu-Tukamuhebwa, R. Nantanda, JB. Jackson, R.J. Blount, E. Wobudeya

**Affiliations:** ^1^ Department of Internal Medicine, University of Iowa, Iowa City, Iowa, USA;; ^2^ Department of Radiology, Mulago National Referral Hospital, Kampala, Uganda;; ^3^ Department of Radiology, University of Iowa, Iowa City, Iowa, USA;; ^4^ Institute for Clinical and Translational Science, University of Iowa, Iowa City, Iowa, USA;; ^5^ Department of Pediatrics, Mulago National Referral Hospital, Kampala, Uganda;; ^6^ Makerere University Lung Institute, Makerere University, Kampala, Uganda;; ^7^ Department of Pathology, University of Iowa, Iowa City, Iowa, USA;; ^8^ MU-JHU Care Ltd/MU-JHU Research Collaboration, Kampala, Uganda.

**Keywords:** tuberculosis, Uganda, spirometry, St. George’s Respiratory Questionnaire, post-TB lung disease

## Abstract

**BACKGROUND:**

The long-term effects of pulmonary TB (PTB) on pediatric lung health are poorly understood. Our objective was to assess predictors of lung function and describe health-related quality of life (QoL) and chest radiograph findings in children following PTB treatment in Kampala, Uganda.

**METHODS:**

We performed a cross-sectional study of children aged 6–16 years who completed PTB treatment within the last five years compared to household controls with no history of active TB. Children underwent chest radiograph, St. George’s Respiratory Questionnaire, and spirometry. Mixed-effects regression models were performed to assess predictors of lung function impairment.

**RESULTS:**

We enrolled 73 children with prior TB and 49 controls. In univariate mixed-effects regression models, prior TB was associated with lower FEV1 and FVC Z-scores (p<0.05). In multivariate models, BMI-for-age Z-score predicted FVC-Z-score (p<0.001). Fibrosis and pleural thickening were common radiographic abnormalities among children with prior TB. Median SGRQ total score was higher among children with prior TB (p<0.001).

**CONCLUSION:**

Children with prior TB showed reduced lung function and QoL compared to household controls. Our findings support the need for routine clinical follow-up after TB treatment.

Each year, approximately 1.3 million children worldwide develop active TB, a disease that remains a leading cause of pediatric illness and mortality in high disease burden countries such as Uganda.^1,2^ Pediatric TB is frequently underdiagnosed and undertreated, and while significant progress has been made in recent years in case detection and treatment, even successfully treated TB can result in pulmonary sequelae.^1,3^ Post-TB lung disease (PTLD) refers to chronic respiratory impairment in individuals treated for pulmonary TB (PTB), with active TB ruled out and no other primary cause of chronic lung disease identified.^4,5^ This definition was proposed at a Post-TB Symposium in 2019 and revised in 2024.^4,5^ PTLD results from the interplay of microbe damage and host immune response, encompassing a range of pathologies including airway obstruction, bronchiectasis, cavitation, fibrosis, or pulmonary vascular disease.^6^ Up to half of adult TB survivors have lung function deficits,^7^ reduced exercise tolerance,^8^ lower quality of life (QoL),^9^ and higher all-cause mortality compared to the general population.^10^

Data on PTLD in children remain limited.^11^ Common respiratory diseases (e.g., bacterial pneumonia), are known to predispose children to lung function deficits and respiratory morbidity in adulthood.^12^ Recent studies in The Gambia and South Africa report that children and adolescents treated for TB are more likely than their peers to experience lung function impairment and reduced exercise capacity.^13,14^ In a recent prospective cohort study, early childhood TB was associated with lung function impairment within the first five years of life.^15^ Due to ongoing lung development, children and adolescents are uniquely susceptible to lung damage. By identifying specific spirometry patterns, radiographic findings, and symptom measures, we can move toward a more standardized, child-specific definition that could be used to guide clinical recommendations and inform potential interventions aimed at reducing the long-term burden of pediatric TB.^16^

Our primary hypothesis is that prior TB is associated with reduced lung function. To test this, we designed and conducted a cross-sectional comparative study to assess predictors of lung function among children who recently completed PTB treatment along with household controls with no history of TB. In addition, we aimed to describe health-related QoL and chest radiographs within the same cohort.

## METHODS

### Study design

We conducted a cross-sectional comparative study of children who completed TB treatment and household controls. The study was based at Mulago National Referral Hospital in Kampala, Uganda, with enrollment occurring from November 2023 to June 2024.

### Study population

Using the National Tuberculosis and Leprosy Program (NTLP) registry, we identified consecutive cases of children aged 6-16 who completed PTB treatment within the past five years. Children under age 6 typically cannot perform spirometry. Previous TB diagnosis followed NTLP guidelines: bacteriologically confirmed TB was defined by positive microscopy, culture, or nucleic acid amplification tests, and clinical diagnosis by negative microbiological test but suggestive symptoms, contact history, and chest radiograph.^17^ Parents were contacted via phone numbers provided in the TB registry, with initial eligibility confirmed by age and proximity to the study site (<50 km). Controls were children aged 6–16 who lived with the child with prior TB for at least half of the TB treatment period. Up to 2 controls per household were recruited to avoid bias from over-representing households, and multiple children with prior TB were recruited from the same household when possible.

### Study visits

The study involved two visits, within 30 days of each other. During the first visit, participants underwent a physical exam, demographic and medical history assessment, TB symptom screening, urine pregnancy testing (for menstruating females only), and chest radiograph. Exclusions included active TB, positive pregnancy test, prior TB (for controls only), and contraindications to spirometry (e.g., recent surgery or respiratory infection). At the second visit, children completed spirometry and St. George’s Respiratory Questionnaire (SGRQ). Medical records for children with prior TB were reviewed to collect TB diagnostic and treatment details.

### Radiographic assessment

Both children with prior TB and controls underwent a chest radiograph as part of TB screening prior to spirometry. Radiographs were independently reviewed by 2 radiologists who were blinded to demographic and clinical information. Following the initial review, both radiologists met to agree to a consensual read for any discordant findings.

### Spirometry

Spirometry was performed following American Thoracic Society (ATS) and European Respiratory Society (ERS) guidelines using calibrated Easy-PC or Pneumotrac spirometers.^18^ Children were coached by the technician. The participants were given inhaled salbutamol, followed by repeat spirometry. Spirometry quality was assessed by a pulmonologist and only children meeting ATS/ERS standards for acceptability and repeatability were included in the analysis.^18^ Z-scores were derived from the race-neutral 2022 Global Lung Initiative (GLI)-reference equations.^19^ Forced expiratory volume in 1 second (FEV1) and forced vital capacity (FVC) were used to assess lung function, with obstruction defined as FEV1/FVC ratio Z-score ≤ -1.645 and restrictive pattern suggested by FEV1/FVC ≥ -1.645 and FVC Z-score ≤ -1.645.^20^ A bronchodilator response was defined as >10% improvement in FEV1 or FVC relative to the predicted value.

### St. George’s Respiratory Questionnaire (SGRQ)

SGRQ is a self-reported questionnaire assessing health-related QoL in patients with chronic obstructive pulmonary disease (COPD).^21^ It includes three subscales (symptoms, activity, and impact) and a total score ranging from 0 to 100, with higher scores representing a decreasing QoL. SGRQ has previously been translated to Luganda.^22^ Two questions were modified for pediatric relevance. Questionnaires were administered to children by trained study nurses, with parents present during administration.

### Statistical analysis

Medians and interquartile ranges (IQR) were used to summarize continuous variables and counts and percentages were used to summarize categorical variables. Comparisons were made by prior TB status using Fisher’s Exact Tests for categorical variables and Wilcoxon rank-sum tests for continuous variables. BMI-for-age (underweight) and height-for-age (stunted) Z-scores were calculated using the 2007 WHO growth reference data.^23^ To test the hypothesis that PTB is associated with lung function, we fitted mixed effects logistic regression models in which a random intercept was included for each household to account for inherent variability between households. FEV1, FVC, and FEV1/FVC ratio Z-scores were the continuous outcomes (each in separate models), with TB as the primary predictor. Univariate models were fit for each outcome, and a multivariable model determined via stepwise selection aimed towards minimizing the Akaike information criterion (AIC) was fit for each outcome. An interaction term between BMI-for-age Z-score and TB status was included as a candidate predictor for the multivariable models to test for effect modification by nutritional status. Statistical significance was defined as p<0.05 for all comparisons. R version 4.4.2 was used for all analyses.

### Ethical considerations

This study was approved by the Mulago Hospital Research and Ethics Committee (MHREC 2023-105), the University of Iowa Institutional Review Board (202304078), and the Uganda National Council for Science and Technology (HS3019ES). Written informed consent was obtained from parents/guardians, with written assent also obtained from children above eight years.

## RESULTS

Seventy-three children were able to be contacted and eligible for enrollment, along with 49 household controls. Eight children with prior TB and five controls were excluded from the spirometry analyses because they did not meet ATS/ERS criteria for acceptability and repeatability. Study selection is described in Figure 1.

**Figure.**
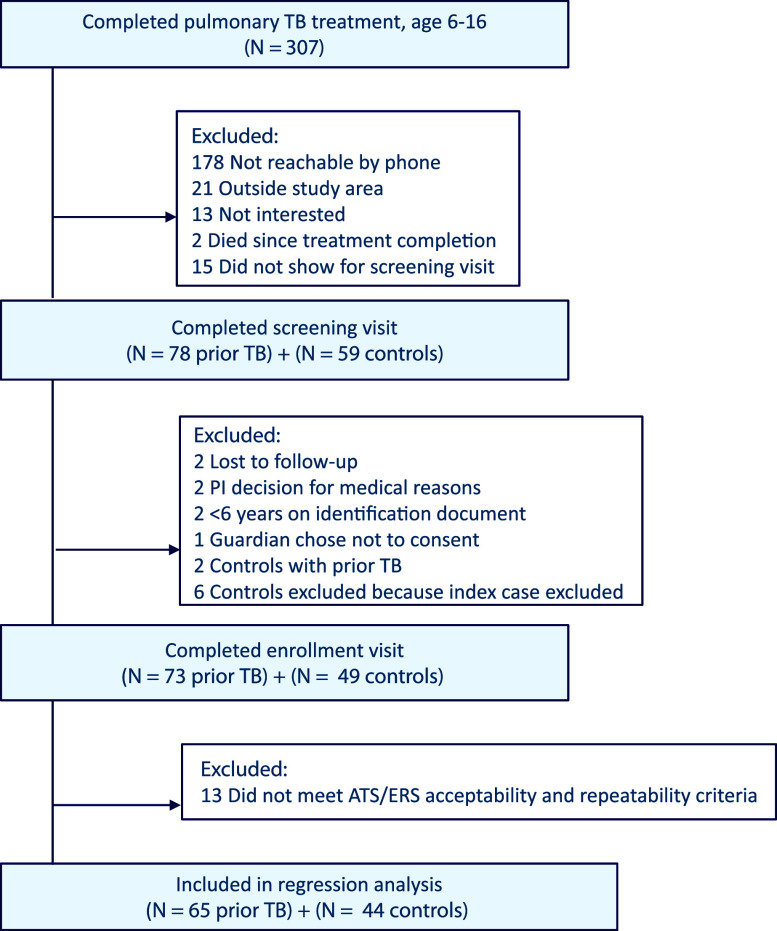
Study flowchart showing selection for screening and enrollment.

Participant demographics and clinical characteristics are shown in Table 1. Children with prior TB had a median age of 9 years (IQR 7, 12) compared to 11 years (IQR 8, 13) in controls (p=0.020). In the prior TB group, 23% were previously diagnosed with bacteriologically confirmed TB, 77% clinically diagnosed, and the median time since completion of TB treatment was 1,001 days (IQR 313, 1452). HIV prevalence was 9.6% in the prior TB group and 0% in controls (p=0.002). BMI-for-age Z-score was lower in the prior TB group (medians -0.26 vs. 0.11, p=0.009), while height-for-age Z-score did not differ (p=0.5). Household characteristics are described in Supplementary Data Table S1. Twenty-four percent of households reported indoor smoking in the prior three months. The primary cooking fuel was charcoal (89%), wood (9.7%), and liquid petroleum gas (1.6%). Eight percent parked a motorcycle indoors in living areas overnight. Forty-five percent of households reported living near a main road.

**Table 1.** Participant demographics and clinical characteristics by prior TB.

	Control N = 49^*^	Prior TB N = 73^*^	p-value^A^
Age in years	11 (8, 13)	9 (7, 12)	0.020
Sex			>0.9
Male	29 (59%)	44 (60%)	
Female	20 (41%)	29 (40%)	
School			>0.9
Day school	41 (84%)	61 (84%)	
Boarding	8 (16%)	12 (16%)	
Not in school	0 (0%)	0 (0%)	
Asthma	0 (0%)	2 (2.7%)	0.5
Days Since TB treatment completion	NA (NA, NA)	1,001 (313, 1,452)	
Disease classification			>0.9
PBC	0 (NA%)	17 (23%)	
PCD	0 (NA%)	56 (77%)	
HIV			0.002
Negative	45 (92%)	66 (90%)	
Positive	0 (0%)	7 (9.6%)	
Do not know	4 (8.2%)	0 (0%)	
BMI-for-age Z-score	0.11 (-0.43, 0.56)	-0.26 (-0.80, 0.18)	0.009
Height-for-age Z-score	-0.12 (-0.71, 0.40)	-0.33 (-1.06, 0.43)	0.5
Cough			0.005
Not at all	18 (37%)	12 (16%)	
Only with chest infections	29 (59%)	43 (59%)	
A few days a month	1 (2.0%)	6 (8.2%)	
Several days a week	0 (0%)	9 (12%)	
Most days a week	1 (2.0%)	3 (4.1%)	
Shortness of breath			0.2
Not at all	42 (86%)	54 (74%)	
Only with chest infections	4 (8.2%)	8 (11%)	
A few days a month	2 (4.1%)	10 (14%)	
Several days a week	1 (2.0%)	0 (0%)	
Most days a week	0 (0%)	1 (1.4%)	
Wheezing			0.12
Not at all	46 (94%)	59 (81%)	
Only with chest infections	3 (6.1%)	8 (11%)	
A few days a month	0 (0%)	5 (6.8%)	
Several days a week	0 (0%)	1 (1.4%)	
Most days a week	0 (0%)	0 (0%)	

* n(%); Median (Quartile 1, Quartile 3);

A Wilcoxon rank sum test; Fisher’s exact test.

PBC = pulmonary bacteriologically confirmed; PCD = pulmonary clinically diagnosed; BMI = body mass index.

Spirometry results comparing children with prior TB to controls are summarized in Table 2. Children with prior TB had lower median FEV1 Z-scores (-0.78 versus -0.52, p=0.012) and FVC Z-scores (-0.82 versus -0.56, p=0.038) compared to controls, but not FEV1/FVC Z-score (p=0.2). A normal spirometry pattern was observed in 95% of controls and 78% of children with prior TB (p=0.014). Obstructive pattern was present in 4.6% of children with prior TB and 0% controls (p=0.3), while restrictive pattern was present in 17% prior TB and 4.5% controls (p=0.070). Chest radiographs were abnormal in 21% of the prior TB group versus 10% of controls (p=0.14) (Supplementary Data Table S2). Among children with prior TB who had abnormal radiographs, the most common findings were fibrosis 9/15 (60%), pleural thickening 5/15 (33%), hilar adenopathy 3/15 (20%), volume loss 2/15 (13%), and calcified granuloma 2/15 (13%). SGRQ results are shown in Table 3. The median total score was 2 (IQR 1, 7) for controls and 10 (3, 30) for children with prior TB (p<0.001). The median symptoms sub-score was 11 (IQR 0, 14) for controls and 21 (IQR 11, 38) for children with prior TB (p<0.001). The median activity sub-score was 0 (IQR 0, 6) for controls and 7 (IQR 0, 30) for children with prior TB (p<0.001), and the median impact sub-score was 0 (IQR 0, 2) for controls and 9 (IQR 2, 26) for children with prior TB (p<0.001).

**Table 2.** Spirometry values among participants by prior TB.

	Control N = 44^*^	Prior TB N = 65^*^	p-value^A^
FEV1 (L)	1.98 (1.42, 2.47)	1.57 (1.30, 2.00)	0.008
FVC (L)	2.16 (1.66, 2.83)	1.76 (1.45, 2.17)	0.012
FEV1/FVC ratio	0.91 (0.85, 0.92)	0.89 (0.84, 0.92)	0.4
FEF25-75 (L/s)	2.59 (1.82, 3.13)	2.16 (1.60, 2.69)	0.011
Z-scores			
FEV1	-0.52 (-0.90, 0.12)	-0.78 (-1.42, -0.37)	0.012
FVC	-0.56 (-0.91, -0.05)	-0.82 (-1.54, -0.22)	0.038
FEV1/FVC ratio	0.26 (-0.54, 0.69)	-0.22 (-0.71, 0.55)	0.2
Spirometry pattern^B^			
Obstructive	0 (0%)	3 (4.6%)	0.3
Possible restriction	2 (4.5%)	11 (17%)	0.070
Normal	42 (95%)	51 (78%)	0.014
Bronchodilator response^C^	3 (6.8%)	7 (11%)	0.7

* n(%); Median (Quartile 1, Quartile 3);

A Wilcoxon rank sum test; Fisher’s exact test;

B Obstructive is defined as FEV1/FVC ratio Z-score ≤ -1.645; Possible Restriction is defined as FEV1/FVC ratio Z-score > -1.645 and a FVC Z-score ≤ -1.645; Normal is defined as a FEV1/FVC ratio Z-score > -1.645 and a FVC Z-score > -1.645;

C Defined as a change of more than 10% in FEV1 or FVC relative to the predicted value.

FEV1 = forced expiratory volume in 1 second; FVC = forced vital capacity; FEV1/FVC ratio = ratio of forced expiratory volume in 1 second to forced vital capacity; FEF25-75 = forced expiratory flow at 25% to 75% of the pulmonary volume.

**Table 3.** St. George’s Respiratory Questionnaire Scores by Prior TB.

	Control N = 49*	Prior TB N = 73*	p-value^A^
Total score	2 (1, 7)	10 (3, 30)	<0.001
Component score			
Symptoms	11 (0, 14)	21 (11, 38)	<0.001
Activity	0 (0, 6)	7 (0, 30)	<0.001
Impacts	0 (0, 2)	9 (2, 26)	<0.001

* Median (Quartile 1, Quartile 3);

A Wilcoxon rank sum test.

In mixed-effect univariate regression models, prior TB corresponded with a 0.56 lower predicted FEV1 Z-score (β -0.56, CI -0.94, -0.19, p=0.004), a 0.41 lower predicted FVC Z-score (β -0.41, CI -0.76, -0.05, p=0.025), and a 0.35 lower predicted FEV1/FVC ratio Z-score (β -0.35, CI -0.66, -0.44, p=0.029) (see Table 4). No significant association was found between the time since TB treatment and lung function in children with prior TB. In multivariate models, the association between TB and FEV1/FVC ratio Z-score persisted (β -0.43, CI -0.77, -0.10, p=0.012). Other significant associations in multivariate models included BMI-for-age Z-score x TB interaction, which predicted a 0.44 higher FEV1 Z-score (β 0.44, CI 0.03, 0.84, p=0.034).

**Table 4.** Mixed effect regression of spirometry.

	Univariate analysis (N=109)			Multivariate analysis (N=109)		
	FEV1 Z-score	FVC Z-score	FEV1/FVC Z-score	FEV1 Z-score	FVC Z-score	FEV1/FVC Z-score
Prior TB	β -0.56 (CI -0.94, -0.19), p=0.004	β -0.41 (CI -0.76, -0.05), p=0.025	β -0.35 (CI -0.66, -0.44), p=0.029	—	—	β -0.43 (CI -0.77, -0.10), p=0.012
BMI-for-age Z-score	β 0.57 (CI 0.37, 0.77), p<0.001	β 0.60 (CI 0.42, 0.79), p<0.001	β -0.08 (CI -0.26, 0.09), p=0.3	—	β 0.58 (CI 0.39, 0.78), p<0.001	β -0.26 (CI -0.44, 0.08), p=0.005
Abnormal chest radiograph	β -0.87 (CI -1.4, -0.38), p<0.001	β -0.73 (CI -1.2, -0.25), p=0.003	β -0.32 (CI -0.73, 0.09), p=0.13	—	—	—
SGRQ total score	β -0.01 (CI 0.03, 0.00), p=0.033	β -0.01 (CI -0.02, 0.00), p=0.14	β -0.01 (CI -0.02, 0.00), p=0.031	—	—	β -0.01 (CI -0.02, 0.00), p=0.040
HIV	β 0.51 (CI -0.34, 1.4), p=0.2	β 0.39 (CI -0.42, 1.2), p=0.3	β 0.28 (CI -0.41, 0.97), p=0.4	—	—	β 0.96 (CI 0.27, 1.6), p=0.007
BMI x TB	—	—	—	β 0.44 (CI 0.03, 0.84), p=0.034	—	—
Days since TB treatment completion^*^	β 0.00 (CI 0.00, 0.00), p=0.2	β 0.00 (CI 0.00, 0.00), p=0.3	β 0.00 (CI 0.00, 0.00), p=0.3	—	—	—

* N=65, limited to children with prior TB.

β **=** beta regression coefficient; BMI = body mass index; CI = Confidence Interval; FEV1 = forced expiratory volume in 1 second; FVC = forced vital capacity; FEV1/FVC ratio = ratio of forced expiratory volume in 1 second to forced vital capacity; SGRQ = St. George’s Respiratory Questionnaire.

## DISCUSSION

In this study, children who had completed PTB treatment within the prior five years had significantly lower spirometry Z-scores compared to household controls with no history of TB. Children with prior TB had reduced QoL, as evidenced by higher SGRQ scores. The most common radiographic abnormalities among children following TB treatment were fibrosis and pleural thickening. TB was predictive of FEV1, FVC, and FEV1/FVC Z-scores but did not predict obstructive or restrictive patterns. The clinical relevance of these findings is supported by higher SGRQ scores among children with prior TB. Given the overall limited data on post-TB sequelae, our findings could reflect excellent lung function outcomes or indicate subtle immunological alterations that may have important long-term implications.^24^ Further longitudinal studies are needed to assess the progression of PTLD over time, especially in youth populations.

Nutrition appeared to have a stronger association with lung function in children with prior TB than controls. This is supported by the positive and significant interaction term between prior TB and BMI-for-age on FEV1 Z-score. Being underweight can result in slow lung growth, reduced alveolarization, and resulting lung function impairment.^25^ Furthermore, TB can worsen malnutrition through increased metabolic demand and alterations in micronutrient metabolism, further impairing lung health.^26,27^ TB treatment has been shown to increase weight and fat mass during the intensive treatment phase,^28^ though in our cohort, children who completed TB treatment still weighed significantly less than household controls.

Restriction was the most common abnormal spirometry pattern among children post-TB, with 17% and 4.6% exhibiting restriction and obstruction, respectively. A previous study in The Gambia reported that 36.4% of children had restriction and 1.9% obstruction at least 6 months following TB treatment, although different reference equations were applied in this study.^14^ Post-TB studies in adults have shown heterogeneous results, with obstruction, restriction, and mixed spirometry patterns.^29^ This variability is interesting and may reflect prolonged exposure to respiratory insults over time, including smoking and air pollution, both of which can lead to the development of COPD.

Children with prior TB had reduced QoL compared to controls across symptoms, impact, and activity sub-scores, and the SGRQ total score was significantly associated with FEV1 and FEV1/FVC Z-scores in regression models. SGRQ has not been validated in children, nor is it commonly used for restrictive lung disease, as the symptom component, including wheezing, is less relevant. A study in The Gambia measured health-related QoL using the PedsQL V4.0, a 23-item questionnaire assessing physical, emotional, social, and school functioning. Children previously treated for TB had lower median scores across almost all domains compared with controls.^14^ As we consider potential interventions for PTLD, like pulmonary rehabilitation, QoL metrics remain an important tool to assess disease burden and treatment response.^30^

To our knowledge, this is one of the first studies to perform chest radiographs in children following TB treatment. Fibrosis and pleural thickening were the most common abnormalities among children post-TB, and abnormal chest radiograph was significantly associated with FVC Z-score in regression models. Computed tomography studies suggest that children with newly diagnosed TB are susceptible to a range of destructive lung damage including cavitation, fibrosis, and bronchiectasis.^31^ Chest radiographs are affordable, accessible diagnostic tools. Limited radiation exposure for a single-view chest radiograph may be justifiable for the benefit of obtaining reliable management guidance for at-risk children during and after TB treatment.

This study had several limitations. First, as a cross-sectional study, causal inferences on the relationship between prior TB and lung function cannot be made. Second, while spirometry can suggest a restrictive defect, plethysmography is needed to confirm it. Third, participants with prior TB were derived from a registry of 307 cases many of whom could not be contacted and may have been in worse health or deceased, potentially underestimating the true prevalence of impairment. Fourth, household controls were selected to account for shared social and environmental conditions. However, we do not know whether the controls and children with prior TB lived together for the entire period between TB treatment and study enrollment. Finally, this study was conducted at a referral hospital in Uganda, and the findings may not be generalizable to other populations.

## CONCLUSION

Children with recent PTB exhibited significantly reduced spirometry Z-scores compared to household controls. Our findings support the use of routine clinical assessment post-TB treatment, including spirometry, to identify at-risk children. Longitudinal studies are needed to understand the progression of chronic lung disease, and interventions are needed to improve long-term outcomes for children with PTLD.

## Supplementary Material


