# Supplementary material for: Post-TB lung function, quality of life, and radiographic findings in children
Source: IJTLD Open. 2025 Jul 9;2(7):427–33. doi: 10.5588/ijtldopen.24.0675 (PMC12248413; doi:10.5588/ijtldopen.24.0675)
Supplement: Supplementary file 1 [file ijtldopen24-0675_supplementarydata1.pdf]

Supplemental Table 1 – Household characteristics and environmental factors

	**N = 62***
**Highest education completed by person living in compound**	
No education	0 (0%)
Primary Incomplete	5 (8.1%)
Primary Complete	5 (8.1%)
Secondary Incomplete	15 (24%)
Secondary Complete	14 (23%)
Post-secondary	23 (37%)
**Number of persons living in compound**	6 (5, 8)
**Indoor smoking in compound in prior three months**	15 (24%)
**Cooking Location**	
Outdoor	28 (45%)
Indoor living areas	11 (18%)
Indoor not in living area	23 (37%)
**Most common cooking fuel**	
Charcoal	55 (89%)
Wood	6 (9.7%)
Liquid Petroleum Gas	1 (1.6%)
**Parking motorcycle indoor overnight**	5 (8.1%)
**Lives on a main road†**	28 (45%)
**If no, walking time (minutes) to nearest main road**	5 (3, 20)

* n (%); Median (Quartile 1, Quartile 3)
† A main road defined as one where you see regular public transport, it is busier, and often used by people from outside your neighborhood or town to get to other places.

Supplemental Table 2 – Chest radiograph abnormalities among participants by prior TB

		**Control** N = 49*	**Prior TB** N = 73*	**p-value**†
**Chest radiograph overall impression**	122			0.14
Normal		44 (90%)	58 (79%)	
Abnormal		5 (10%)	15 (21%)	
**Findings**	20			
Fibrosis		2 (40%)	9 (60%)	0.6
Pleural thickening		0 (0%)	5 (33%)	0.3
Hilar adenopathy		1 (20%)	3 (20%)	>0.9
Volume loss		0 (0%)	2 (13%)	>0.9
Calcified granuloma		0 (0%)	2 (13%)	>0.9
Blunted angle		0 (0%)	1 (6.7%)	>0.9
Consolidation		1 (20%)	0 (0%)	0.3
Bronchiole dilation		0 (0%)	1 (6.7%)	>0.9
Atelectasis		0 (0%)	1 (6.7%)	>0.9
Hyperaeration		1 (20%)	0 (0%)	0.3

* n (%)

† Fisher’s exact test
